# Com probe implemented STexS II greatly enhances specificity in SARS-CoV-2 variant detection

**DOI:** 10.1038/s41598-022-24530-w

**Published:** 2023-01-19

**Authors:** Jae Jong Kim, Hyoung-Min Park, A. Young Kyoung, Si-Kyu Lim, Sun Ho Cha, J. Eugene Lee, Byoung Chul Park

**Affiliations:** 1 GenoTech Corporation, 26-69, Gajeongbuk-ro, Yuseong-gu, Daejeon, 34113 Republic of Korea; 2 Biometrology Group, Korea Research Institute of Standards and Science, 267 Gajeong-ro, Yuseong-gu, Daejeon, 34113 Republic of Korea; 3 Critical Diseases Diagnostics Convergence Research Center, Korea Research Institute of Bioscience and Biotechnology, Daejeon, 34145 Republic of Korea

**Keywords:** Biological techniques, Diseases

## Abstract

The initial introduction of utilizing double helix structural oligonucleotides known as SNP typing with excellent specificity (STexS) in a standard PCR greatly improved the detection of single nucleotide polymorphisms (SNP) by enhancing amplification rates of primer-matching strands and interrupting mismatched strands by constant instability of kinetics regarding alignment attaching and detaching. The model was beneficial overall in detecting SNP variants consisting of large amounts of wildtype strands such as EGFR mutation genotyping for early detection of non-small cell lung cancer. While the STexS PCR is advantageous in detecting SNPs and biomarkers, limitations were yet observed. Despite the ability to detect variants 10 times more effective than a typical amplification-refractory mutation system PCR, it could only perform optimally in DNA concentrations around 101 ~ 105. To further enhance STexS specificity to perform detecting viral-RNA variants such as the infamous SARS-CoV-2, a novel improvement of the regular TaqMan Probe using Com-probes to inhibit high copy wild targets and amplify low copy mutant targets. By introducing the novel STexS II, omicron variants of SARS-CoV-2 were able to be successfully detected in high concentrations of normal genes.

## Introduction

Detection of genetic alterations such as SNPs is crucial in tackling various genetic disorders and cancers. As research revealed genetic disorders and various cancers can be triggered by a single nucleotide mutation, the importance of distinguishing such diseases through methods of genotyping continues^1,2^. Currently, the dominant concept in locating SNP is through PCR. Amplification of highly specific primers matching to certain mutated genomic regions results in time-efficient and cost-effective results that are relatively reliable^3^. To further increase specificity, various implements are added to the PCR process. As the basis of PCR is separated into three steps; denaturation, annealing, and extension, improvements were observed in several aspects^4^. Supplements such as probes are designed to give fluorescent signals during annealing, enabling comparative quantitative analysis that led to qPCR^5^. Other approaches focused on customizing primer sequences to obtain highly specific matches and increasing the annealing temperature to block potential dimers^6^. The recently introduced SNP typing with excellent specificity (STexS) model also focuses on the annealing process. By implementing double helix structured oligonucleotides within the primer, small portions of matched primers were successfully discriminated from the abundant mismatched templates^7^. All the methods mentioned above eventually benefit the overall PCR process to detect variants.

While the STexS platform was able to perform outstandingly in a genomic environment, limitations were soon to be seen when implemented in a viral circumstance. Among the varieties of the virus, SARS-CoV-2 has been the dominant threat to public health. The ability to disseminate worldwide and inflict high casualties during a short period is concerning^8^. To make matters worse, mutated variants of SARS-CoV-2 continue to derail the overall effort to effectively diagnose and suppress^9^. The Coronaviridae is an RNA virus replicating by reverse transcription followed by DNA replication and transcription. The extended replication process enables the virus to have more alterations of the original template which then can form characteristics not seen in previous infections^9^. This gradually led to distinct variants known as Alpha, Beta, Gamma, Delta, and Omicron. While the other four subtypes have been relatively subdued through various types of vaccines, the Omicron variant continues to spread among the population. Reports indicate SARS-CoV-2 can be detected via RT-PCR 2 to 3 days prior to symptoms being observed^10^. Further reports show the virus to have a high transmission rate within 1 ~ 2 week after the initial exposure^11^. To cause the SARS-CoV-2 to further diminish, effective detection methods that can discriminate infected strands within yet inflicted strands are essential.

To achieve such goals, this study focused on developing the previous STexS method to effectively detect and amplify mutated regions of SARS-CoV-2 while simultaneously diminishing unnecessary strands by inhibiting signal emission to increase specificity. As a result, we introduce an enhanced method utilizing a novel probe mixture named Combination and Competition probes (Com probes).

## Results

### qPCR enhancements by utilizing Com probes

**Figure 1:**
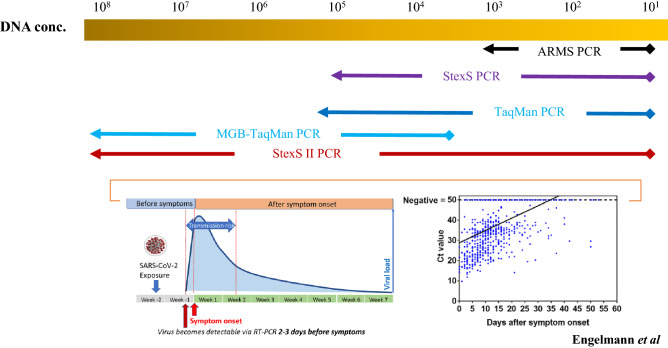
Comparison between methods of PCR viable in various DNA concentrations and characteristics of SARS-CoV-2 as an RNA-based environment. Clinical significance of SARS-CoV-2 RT-PCR results adapted from Reanalytical Issues and Cycle Threshold Values in SARS-CoV‑2 Real-Time RT-PCR Testing: Should Test Results Include These? By Ilka Engelmann et al. (2021), ACS Omega 6 (10), 6528–6536.

Unlike cell-based DNA genotyping, viral SNP, especially RNA-based viridae, consists of wide concentrations ranging from 10^1^ to 10^8^ copies. The typical ARMS PCR was able to detect variants within the 10^1^–10^3^ range. STexS was able to improve the detection range to 10^1^–10^5^. Despite the enhanced specificity, viral variants could be seen in far concentrated amounts that exceed the current detection limit. To overcome the challenge and increase specificity, methods such as TaqMan and minor groove binder (MGB) included TaqMan probes were introduced for distinct purposes^12^. While the original Taqman probe focused on normalizing and quantifying relatively small concentrations around 10^1^–10^5^, MGB included TaqMan probes that tend to perform optimally in high concentrations around 10^4^–10^8^. To detect variants of RNA-based viruses such as SARS-CoV-2, the spectrum must be broadened that envelops small and high concentrations^13^. As a result, the concept of STexS II was based on novel TaqMan probe enhancements (Fig. 1).

**Figure 2:**
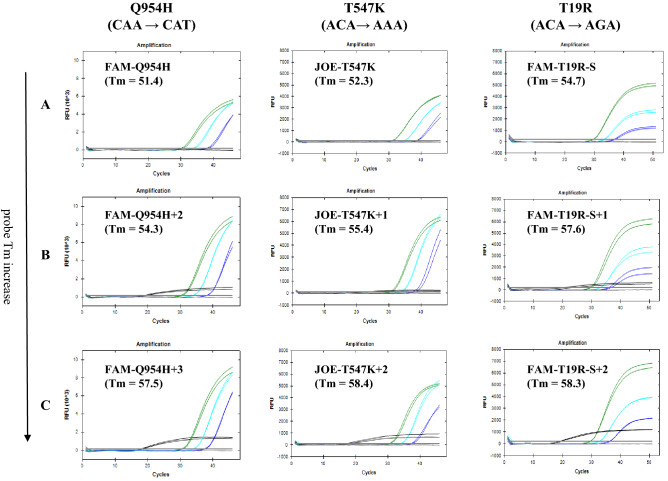
Correlation between probe Tm increase and PCR signal enhancement. Each Probe targets the omicron mutated region Q954H, T547, and Delta mutated region T19R. The Tm of each probe was adjusted by adding one base in the 3’ end per model ((**A**): Original template, (**B**): 1 ~ 2 bases added, (**C**): 2 ~ 3 bases added).

In the previous research, one of the priorities of optimal signal amplification was adjusting reaction rates and melting temperatures (Tm) to be above the lowest annealing temperature to rule out unnecessary dimers^7^. Similarly, the correlation between probe Tm and PCR signal elevation needed to be observed. By targeting the main mutation sites of the omicron variant Q954H, T547K, and delta variant T19R, the overall PCR signal was correlated with probe Tm increase (Fig. 2). Further elongating the probe sequence resulted in a gradual increase of an average 3 °C per base alongside a higher increase in mutant amplification. Despite the increased amplification rates, the increase was also observed in wildtype strands leading to a decrease in cycle threshold (Ct) value when quantified (Sup Table 1). To block unwanted signals from amplifying, the probe sequence alone needed to be shortened, forfeiting the advantages of having high specificity in the detection of low-copy targets (Table 1). To use an extended probe and block WT signals, a different approach was inevitable.

**Table 1:** Probe sequence and Tm information used for correlation.

Target	Probe Name		Sequence	Tm (°C)
Q954H (CAA → CAT)	A	FAM-Q954H	5’-FAM-TGGTCAAC**CAT**AATGCAC-BHQ1	51.4
	B	FAM-Q954H + 2	5’-FAM-TGGTCAAC**CAT**AATGCACAA-BHQ1	54.3
	C	FAM-Q954H + 3	5’-FAM-TGGTCAAC**CAT**AATGCACAAG-BHQ1	57.5
T547K (ACA → AAA)	A	JOE-T547K	5’-JOE-TCAATGGTTTA**AAA**GGCACA-BHQ1	52.3
	B	JOE-T547K + 1	5’-JOE- TCAATGGTTTA**AAA**GGCACAG-BHQ1	55.4
	C	JOE-T547K + 2	5’-JOE- TCAATGGTTTA**AAA**GGCACAGG-BHQ1	58.4
T19R (ACA → AGA)	A	FAM-T19R-S	5’-FAM- TGTGTTAATCTT**AGA**ACCAGAA -BHQ1	54.7
	B	FAM-T19R-S + 1	5’-FAM- TGTGTTAATCTT**AGA**ACCAGAAC-BHQ1	57.6
	C	FAM-T19R-S + 2	5’-FAM-TGTGTTAATCTT**AGA**ACCAGAACT-BHQ1	58.3

**Figure 3:**
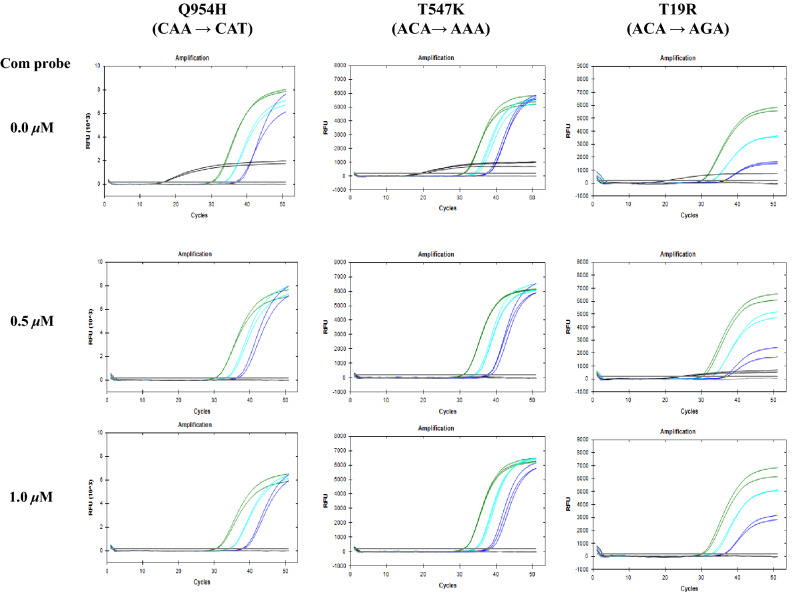
Diminish of nonspecific signals by utilizing Com probes. Multiplex probes consist of elongated probes 2 ~ 3 bases added from the original probe and Com probes. Each PCR trial is performed in triplicates.

Combining several probes within a single PCR is not a novel concept. A typical probe combination consists of probes emitting different colors which attach to different primers^14^. This condenses the PCR workflow to quantify several targets simultaneously. The multiplex method of probes was possible as probes were designed to react independently with no interference within each group^15^. While this is the main approach for using multiplex probes, the STexS II Com probes were implemented to interfere with and block nonspecific signals, gradually increasing the gap between mutated and nonspecific signals. Each Com probe was designed to attach wildtype regions that parallel to the mutated sequence (Table 2). For instance, the FAM-Q954H probe and its Com probe target the identical sequence of the Q954H region. While the Q954H probe targets the mutated sequence, the Com probe of Q954H targets the unaltered sequence. Once the probes are activated, the mutated target signals will be attached to target probes and initiate amplification, while the nonspecific strand will compete in its binding phase between the Q954H and Com probe. Initially, the improved STexS II method was tested whether it improves the previous dynamic range. As a result, STexS II successfully detected low (10^2^) and high (10^8^) concentrations (Sup Fig. 1). After validation, surprisingly, Com probes drastically decreased the amplification of nonspecific signals of Q954H, T547K, and T19R regions (Fig. 3). Because the Com probe has a similar structure against the target probe, further analysis for finding signs of probe interference was performed. When the Ct value of each target was measured, Com probes did not hinder the overall quantification in a significant manner (Sup Table 2). As a result, mutation detection of the omicron variant was successful using probes in a combinative and competitive concept.

**Table 2:** Correlation between Com probe and nonspecific signal and Ct value changes.

		probe (μM)	Com probe (μM)	Mutant 5 × 10^3^	Mutant 5 × 10^2^	Mutant 5 × 10^1^	WT 5 × 10^7^
Q954H (CAA → CAT)	ⓐ	0.75	0	30.11	33.54	37.02	16.53
	ⓑ	0.75	0.50	30.26	33.30	36.44	0.00
	ⓒ	0.75	1.00	30.55	34.30	38.05	0.00
T547K (ACA → AAA)	ⓐ	0.75	0	30.95	33.88	37.65	19.09
	ⓑ	0.75	0.50	30.81	34.12	37.47	0.00
	ⓒ	0.75	1.00	31.08	34.09	37.57	0.00
T19R (ACA → AGA)	ⓐ	0.75	0	30.09	32.88	36.86	20.57
	ⓑ	0.75	0.50	29.97	32.82	36.67	24.66
	ⓒ	0.75	1.00	30.27	32.89	36.33	0.00

**Figure 4:**
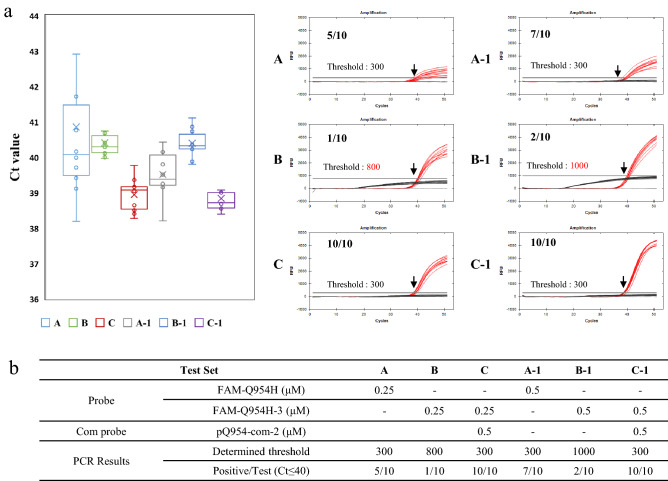
STexS II validation of the Com probe as a viable method for effective positive detection. Validation of signal enhancements and nonspecific signal diminish by Com probes. (**a**) PCR and Ct value of Omicron region Q954H. Each trial was performed 10 times for statistical comparison. (**b**) Information regarding the above PCR trial and results of positive detection.

For further validation and optimization, multiple trials regarding STexS II were performed adjusting the amount of probes and probe sequences. Analyzing multiple trials of PCR targeting Q954H, probes that are elongated amplified a higher Ct threshold compared to the original template (Fig. 4a). The overall enhanced specificity did affect the increase in target amplification. When accompanied by Com probes, the signal of nonspecific strands was mostly diminished, resulting in mostly positive detections (Fig. 4b). Further increasing the usage of probes and Com probes increased the overall signal threshold, but since the previous Ct value was high enough for positive results, even a small volume of Com probes was sufficient for effective PCR detection.

**Figure 5:**
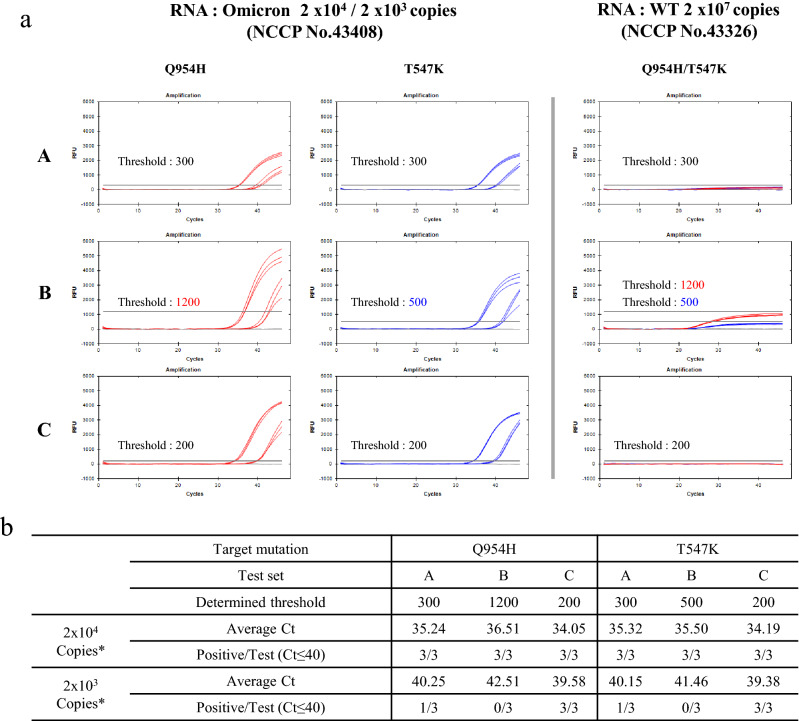
STexS II validation using standard RNA samples of the SARS-CoV-2 Omicron variant. Validation of signal enhancements and nonspecific signal diminish by Com probes within Omicron variant RNA samples. (**a**) PCR and Ct value of Omicron region Q954H. Each trial was performed 3 times for statistical comparison. PCR experimental order is identical to Fig. 4. (**b**) Information regarding the above PCR trial and results of positive detection.

Once the potential of Com probes was observed, RNA samples of omicron variants were further tested whether the novel STexS II will be effective in lower copies. Three trials of PCR targeting the omicron mutation site Q954H and T547K were performed with identical conditions applied in Fig. 4 (Sup Table 3). When comparing PCR detection with or without the use of Com probes in high copies of 2 × 10^4^, all methods were able to successfully result in positive detection (Fig. 5). However, when the RNA copy was decreased, only the PCR using Com probes with elongated probes was able to yield positive detection due to increased specificity alongside diminished amplification of nonspecific strands. The results confirm that STexS II can perform not only in high concentrations of RNA but also in low densities.

## Discussion

**Figure 6:**
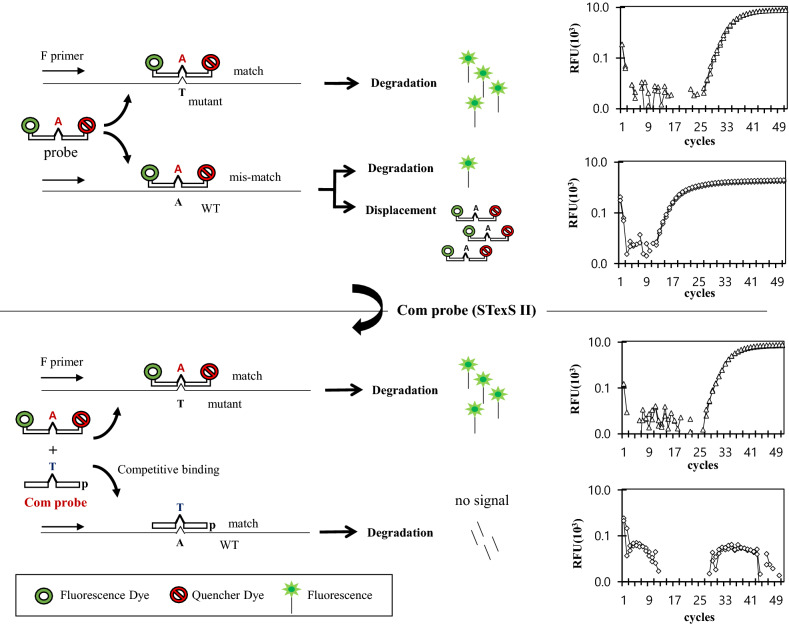
Schematics of WT signal inhibition by STexS II.

For PCR genotyping to successfully detect mutation, not only the ability to efficiently bind to the target region is crucial, but also the difference between target strands and nonspecific strands needs to be significant to consider as a positive result. Hence the detection will still be viable even though the actual Ct value of targets would be low due to diminished amplification rates of nonspecific strands. Typical PCR methods overcome such obstacles by using short probes to block any unwanted binds. However, using relatively short probes would lead to two major disadvantages: the low specificity due to low Tm and low amplification rates of target strands not having a stable bind during alignments. The low amplification rate will eventually mean the method will not be viable in low concentrations and RNA targets such as SARS-CoV-2 detection will be a challenge. Substituting probes with longer sequences indeed increases the amplification of targeted templates but companies with an increase of WT signals. Despite the probe and WT having mismatches, a small portion of the abnormal bond proceeds degradation resulting in a traceable signal hindering the overall Ct value. The introduced STexS II overcomes the problem by implementing an elongated main probe that effectively amplifies the target strand and a secondary probe that competes with the main probe in nonspecific binding. The normal concept of probes is to successfully match to templates and emit detectable signals through fluorescence. However, Com probes shift the approach by intentionally binding to WT strands without hydrolyzable dyes. As the Com probe perfectly matches with the WT strand, no further displacements will occur once it is matched, further decreasing the main probe to have mismatches. When the Com probe couples with WT strands, the actual amplification cannot be quantified since no fluorescence is detected after degradation (Fig. 6). Therefore, the STexS II platform can effectively yield positive detection by increasing the gap between high specific target amplification and untraceable nonspecific signals. The method further discriminates the target region of the omicron variant in both high and low concentrations.

By achieving such an effective method, the STexS II can be implemented on various occasions. Whether it would be a DNA or RNA environment, high or low concentrated copy, using the novel concept of combining probes in a competitive manner will successfully perform in detecting mutation. Also despite the constant mutation leading to a novel SARS-CoV-2 variant inevitable, STexS II will continue to provide an effective method to further detect mutations and overall understanding for future diagnosis.

## Materials and methods

All methods were performed and approved under the supervision and guidelines of the Korean Research Institute of Bioscience and Biotechnology (KRIBB) and the Korean National Culture Collection for Pathogens (NCCP). Both institutes are government affiliations that confirm integrity and regulations.

### Primer and probe design

Each primer was designed and manufactured to match the omicron variant mutation location Q954H and T547K. Probes were designed and manufactured to match above mentioned primers. Probe elongation was performed by adding one base to the 3' end. A Series of probes elongation was tested for optimal performance and Tm adjustments. Com probes were designed to not emit any fluorescent color only to act as a competitive combination in binding nonspecific probes. The sequence of Com probes was designed to be one base different from the main probe.

### Quantitative PCR

Each qPCR was performed consisting of forward and reverse primers matching the Q954H and T547K region SARS-CoV-2. For quantitative measures, the main hydrolyzable fluorescent probes were attached with a FAM or JOE fluorescence in the 5' end for Fluorescence Resonance Energy Transfer (FRET) and a BHQ1 quencher in the 3' end. Com probes were added an additional phosphate in the 3' end to inhibit DNA polymerases to attach and amplify. The buffer solution of each PCR was calculated to total 20 μL using Enzynomics™ solution (cat no. RT 431M). The PCR was performed within 45 ~ 50 cycles after a 10 min reaction in 95 °C, with each cycle consisting of 10 ~ 15 s in 95 °C and 10 ~ 15 s in 60 °C in the CFX9600 Real-Time System (Biorad™). Omicron variant standard samples were performed with the same PCR platform.

### Omicron variant standard sample

RNA samples of omicron variants of SARS-CoV-2 were obtained publicly through the National Culture Collection for Pathogens (NCCP). Three sample collections NCCP43326, NCCP43410, and NCCP43408 were stored for further STexS II trials.

## Supplementary Information

Supplementary Information 1.

Supplementary Information 2.

Supplementary Legends.

## Data Availability

The datasets used and/or analyzed during the current study are available from the corresponding author upon reasonable request.

## References

[1] Kim S, Misra A (2007). SNP genotyping: technologies and biomedical applications. Annu. Rev. Biomed. Eng..

[2] Shastry BS (2007). SNPs in disease gene mapping, medicinal drug development and evolution. J. Hum. Genet..

[3] Myakishev MV (2001). High-throughput SNP genotyping by allele-specific PCR with universal energy-transfer-labeled primers. Genome Res..

[4] Garibyan L, Avashia N (2013). Polymerase chain reaction. J. Invest. Dermatol..

[5] VanGuilder HD, Vrana KE, Freeman WM (2008). Twenty-five years of quantitative PCR for gene expression analysis. Biotechniques.

[6] Liu C (2019). Development of a blocking primer to inhibit the PCR amplification of the 18S rDNA sequences of *Litopenaeus vannamei* and its efficacy in *Crassostrea hongkongensis*. Front. Microbiol..

[7] Kim JJ (2021). Inclusion of double helix structural oligonucleotide (STexS) results in an enhance of SNP specificity in PCR. Sci. Rep..

[8] Tay MZ (2020). The trinity of COVID-19: Immunity, inflammation and intervention. Nat. Rev. Immunol..

[9] Harvey WT (2021). SARS-CoV-2 variants, spike mutations and immune escape. Nat. Rev. Microbiol..

[10] Mallett S (2020). At what times during infection is SARS-CoV-2 detectable and no longer detectable using RT-PCR-based tests? A systematic review of individual participant data. BMC Med..

[11] Mahmood M (2021). Transmission frequency of COVID-19 through pre-symptomatic and asymptomatic patients in AJK: A report of 201 cases. Virol. J..

[12] Holland PM (1991). Detection of specific polymerase chain reaction product by utilizing the 5'––3' exonuclease activity of *Thermus aquaticus* DNA polymerase. Proc. Natl. Acad. Sci. U. S. A..

[13] Engelmann I (2021). Preanalytical issues and cycle threshold values in SARS-CoV-2 real-time RT-PCR testing: Should test results include these?. ACS Omega.

[14] Huang Q (2011). Multicolor combinatorial probe coding for real-time PCR. PLoS ONE.

[15] Sun Z (2017). Development of a multiplex probe combination-based one-step real-time reverse transcription-PCR for NA subtype typing of avian influenza virus. Sci. Rep..

