# Supplementary material for: Com probe implemented STexS II greatly enhances specificity in SARS-CoV-2 variant detection
Source: Sci Rep. 2023 Jan 19;13:1036. doi: 10.1038/s41598-022-24530-w (PMC9850334; doi:10.1038/s41598-022-24530-w)
Supplement: Supplementary file 1 — Supplementary Information 1. [file 41598_2022_24530_MOESM1_ESM.pptx]

## Slide 1
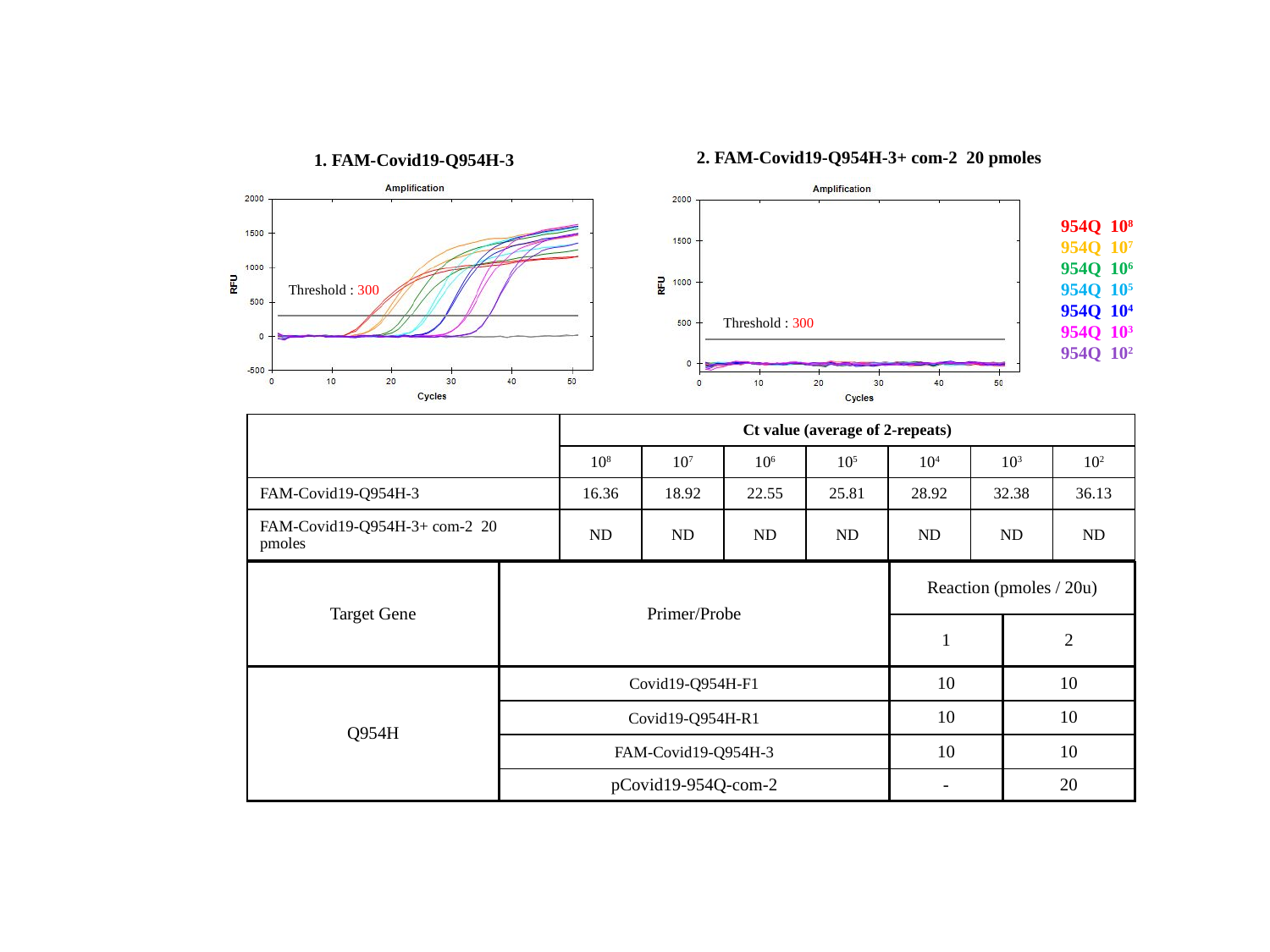

2. FAM-Covid19-Q954H-3+ com-2 20 pmoles
1. FAM-Covid19-Q954H-3
954Q 108
954Q 107
954Q 106
954Q 105
954Q 104
954Q 103
954Q 102
Threshold : 300
Threshold : 300
| | Ct value (average of 2-repeats) | | | | | | |
| --- | --- | --- | --- | --- | --- | --- | --- |
| | 108 | 107 | 106 | 105 | 104 | 103 | 102 |
| FAM-Covid19-Q954H-3 | 16.36 | 18.92 | 22.55 | 25.81 | 28.92 | 32.38 | 36.13 |
| FAM-Covid19-Q954H-3+ com-2 20 pmoles | ND | ND | ND | ND | ND | ND | ND |
| Target Gene | Primer/Probe | Reaction (pmoles / 20u) | |
| --- | --- | --- | --- |
| | | 1 | 2 |
| Q954H | Covid19-Q954H-F1 | 10 | 10 |
| | Covid19-Q954H-R1 | 10 | 10 |
| | FAM-Covid19-Q954H-3 | 10 | 10 |
| | pCovid19-954Q-com-2 | - | 20 |
