# Supplementary material for: Com probe implemented STexS II greatly enhances specificity in SARS-CoV-2 variant detection
Source: Sci Rep. 2023 Jan 19;13:1036. doi: 10.1038/s41598-022-24530-w (PMC9850334; doi:10.1038/s41598-022-24530-w)
Supplement: Supplementary file 2 — Supplementary Information 2. [file 41598_2022_24530_MOESM2_ESM.docx]

Supplementary Table 1. **Cycle threshold changes effected by probe’s enhanced Tm.**

Each Cycle threshold (Ct) value is calculated based on the contrast between the probe amplification against nonspecific strands.

| Probe | Ct (threshold 200), Mutant 5x10^1^ | | |
| --- | --- | --- | --- |
|  | Q954H | T547K | T19R |
| Low Tm probe (A) | 38.08 | 39.20 | 36.80 |
| Medium Tm probe (B) | 38.18 | 38.37 | 36.31 |
| High Tm probe (C) | 37.48 | 37.96 | 36.28 |

Supplementary Table 2. Sequence and Tm information of probes and Com probes used for Figure 3.

| Target | Probe Name | | Sequence | Tm(℃) |  |  |
| --- | --- | --- | --- | --- | --- | --- |
| Q954H  (CAA → CAT) | Probe | FAM-Q954H+3 | 5’-FAM-TGGTCAAC**CAT**AATGCACAAG-BHQ1 | 57.5 | |  |
|  | Com probe | Q954H Combi 2 | 5’-GTGGTCAAC**CAA**AATGCACAAGGC**p** | 65.2 | | |
| T547K  (ACA→ AAA) | Probe | JOE-T547K+2 | 5’-JOE- TCAATGGTTTA**AAA**GGCACAGG-BHQ1 | 58.4 | | |
|  | Com probe | T547K Combi | 5’- TCAATGGTTTA**ACA**GGCACAGGTGp | 63.6 | | |
| T19R  (ACA → AGA) | Probe | FAM-T19R-S+2 | 5’-FAM- TGTGTTAATCTT**AGA**ACCAGAACT | 58.3 | | |
|  | Com probe | T19R-S Combi | 5’- GTGTGTTAATCTT**ACA**ACCAGAACTCAp BHQ1 | 63.7 | | |

Supplementary Table 3. Primer and Probes used for Figure 5

| Target Gene | Primer/Probe | μM | | |
| --- | --- | --- | --- | --- |
|  |  | A | B | C |
| Q954H | Covid19-Q954H-F1 | 0.5 | 0.5 | 0.5 |
|  | Covid19-Q954H-R1 | 0.5 | 0.5 | 0.5 |
|  | FAM-Q954H | 0.5 | - | - |
|  | FAM-Q954H-3 | - | 0.5 | 0.5 |
|  | 954Q-combi 2 | - | - | 0.5 |
| T547K | Covid19-T547K-F | 0.5 | 0.5 | 0.5 |
|  | Covid19-T547K-R | 0.5 | 0.5 | 0.5 |
|  | JOE-T547K+1 | 0.5 | - | - |
|  | JOE-T547K+2 | - | 0.5 | 0.5 |
|  | 547T-combi | - | - | 0.5 |
