# Supplementary material for: Com probe implemented STexS II greatly enhances specificity in SARS-CoV-2 variant detection
Source: Sci Rep. 2023 Jan 19;13:1036. doi: 10.1038/s41598-022-24530-w (PMC9850334; doi:10.1038/s41598-022-24530-w)
Supplement: Supplementary file 3 — Supplementary Legends. [file 41598_2022_24530_MOESM3_ESM.docx]

Supplementary Figure 1. **Correlation between copy number and Ct value changes within STexS Ⅱ**
